# Se-atlas-cartographic representation of experts for rare diseases

**DOI:** 10.1186/1750-1172-9-S1-P1

**Published:** 2014-11-11

**Authors:** Holger Storf, Tobias Hartz, Wulf Pfeiffer, Kathrin Rommel, Mareike Derks, Elisabeth Nyoungui, Joerg Schmidtke, Holm Graessner, Mirjam Knoell, Thomas Wagner, Frank Ueckert

**Affiliations:** 1 Institute for Medical Biostatistics, Epidemiology and Informatics (IMBEI), Obere Zahlbacher Str. 69, 55131 Mainz, Germany; 2 Orphanet Germany, Institute of Human Genetics, Hannover Medical School (MHH), Carl-Neuberg-Str. 1, 30625 Hannover, Germany; 3 Center for Rare Diseases Tübingen, Tübingen University Hospital, Calwerstr. 7, 72076 Tübingen, Germany; 4 Frankfurt Reference Center for Rare Diseases (FRZSE), Frankfurt University Hospital, Frankfurt University Hospital, Theodor-Stern-Kai 7, 60590 Frankfurt, Germany

## Motivation

Numerous highly specialized facilities for the diagnosis and treatment of rare diseases exist in Germany, but it is challenging to present them adequately. Searching in the internet is a common way for patients to find medical information or appropriate clinical experts [1]. Existing services and databases such as Orphanet already offer good contact information to expert centers. A core challenge is to keep the data up to date and to ensure the expertise of the listed centers.

## Aim of the project

The project’s aim is to visualize and present the known expert centers in an innovative way and to help completing existing data bases. The representation is realized in an interactive map and in a list format. Stakeholders of the information system are patients and their relatives, doctors, non-medical personnel and the general public. The basis for the underlying data set of the eligible facilities is provided by the project partner Orphanet Germany. The project’s primary goals are to consistently increase the data set and to ensure its quality.

## Conception

In se-atlas general information about the expert centers like name, address, special consultation hours, etc. are stored. To find the relevant centers, they are tagged additionally with the treated diagnoses (by using the Orphanet classification and ICD-10). The decision which centers will be listed or not will be made by an editorial team based on the so-called NAMSE criteria proposed in the German national plan by the *Joint Declaration and Agreement on the Establishment of the National Action League for People with Rare Diseases* (NAMSE). Both, centers for rare diseases and patient organizations will be allowed to grant affirmations of quality. The conception allows for individual views of the cartographic visualization to be included on other web pages, e.g. those of patient organizations.

## Realization and test

At present, the system is implemented in a prototypical way and accessible for registered persons only. The core functions are realized and the Orphanet classification is included. The next steps are to include the Orphanet data base of expert centres and contact the centers for rare diseases for including them.

## Schedule

*Se-atlas* started in June 2013 and is scheduled to run for two years. The public release of the official version is planned for January 2015.
